# A Rare Presentation of Dentigerous Cyst

**DOI:** 10.7759/cureus.26098

**Published:** 2022-06-19

**Authors:** Sagar S Gaurkar, Prasad T Deshmukh, Chandra Veer Singh, Farhat Q Khan

**Affiliations:** 1 Otolaryngology-Head and Neck Surgery/Surgical Oncology, Jawaharlal Nehru Medical College, Wardha, IND; 2 Otolaryngology-Head and Neck Surgery, Jawaharlal Nehru Medical College, Wardha, IND

**Keywords:** functional endoscopic sinus surgery, fess, follicular cyst, odontogenic cyst, maxillary sinus, dentigerous cyst

## Abstract

Dentigerous cysts are benign, slow-growing odontogenic cysts that are considered to be developmental in origin. They are twice as common in men than in women, and most often occur in people between the ages of 20 and 40 years. They are rarely found in young children. They almost exclusively occur in permanent dentition and over 70% of total number of cases are located in the mandible. Dentigerous cysts most commonly involve the mandibular third molar and are infrequent in maxillary canines. They can grow to a considerable size causing painless expansion of the jaw leading to its deformity.

We report a rare and challenging case of ectopically erupted massive dentigerous cyst of maxillary canine in the anterolateral wall of left maxilla leading to facial deformity and shed a light on its surgical management by the endonasal endoscopic approach in a female pediatric patient.

## Introduction

Dentigerous cysts are benign, slow-growing odontogenic cysts that are considered to be developmental in origin. They are more than twice common in males than in females and frequently occur in individuals between 20 and 40 years of age and are seldom discovered in young children [1,2].

They almost exclusively occur in permanent dentition and over 70% of total number of cases are located in the mandible. The most involved teeth in descending order of occurrence are the mandibular third molars, maxillary third molar, maxillary canine, and mandibular second premolars [3]. Dentigerous cysts related to impacted supernumerary or ectopically erupted tooth localized within the anterior maxilla are very rare and account for only 5.5% of all the cases, while those that extend into the maxillary sinus are even more infrequent [4].

A sine qua non for the development of the cyst is the hydrostatic force exerted by the accumulation of fluid between reduced enamel epithelium and the tooth crown of unerupted teeth which encloses the crown and is attached to the neck at the cementoenamel junction [1]. Dentigerous cysts can expand to an enormous size, and the larger cysts may be accompanied with painless jaw expansion in the involved region. Such extensive lesions can cause facial asymmetry.

We report a rare and challenging case of ectopically erupted massive dentigerous cyst of maxillary canine in the anteromedial wall of left maxilla leading to facial deformity and shed a light on its surgical management by the endonasal endoscopic approach in a female pediatric patient.

## Case presentation

A 12-year-old female was referred to our hospital with a complaint of left facial swelling adjacent to the nose in the nasofacial region, of two years duration, painless, and was gradually increasing in size. The child was initially treated with a course of antibiotics at the previous hospital. Following unresponsiveness of the swelling to the initial medical treatment, an x-ray paranasal sinus was advised which revealed a radiopaque mass in the left maxillary sinus near the floor of the orbit with cloudy appearance of the sinus.

**Figure 1:**
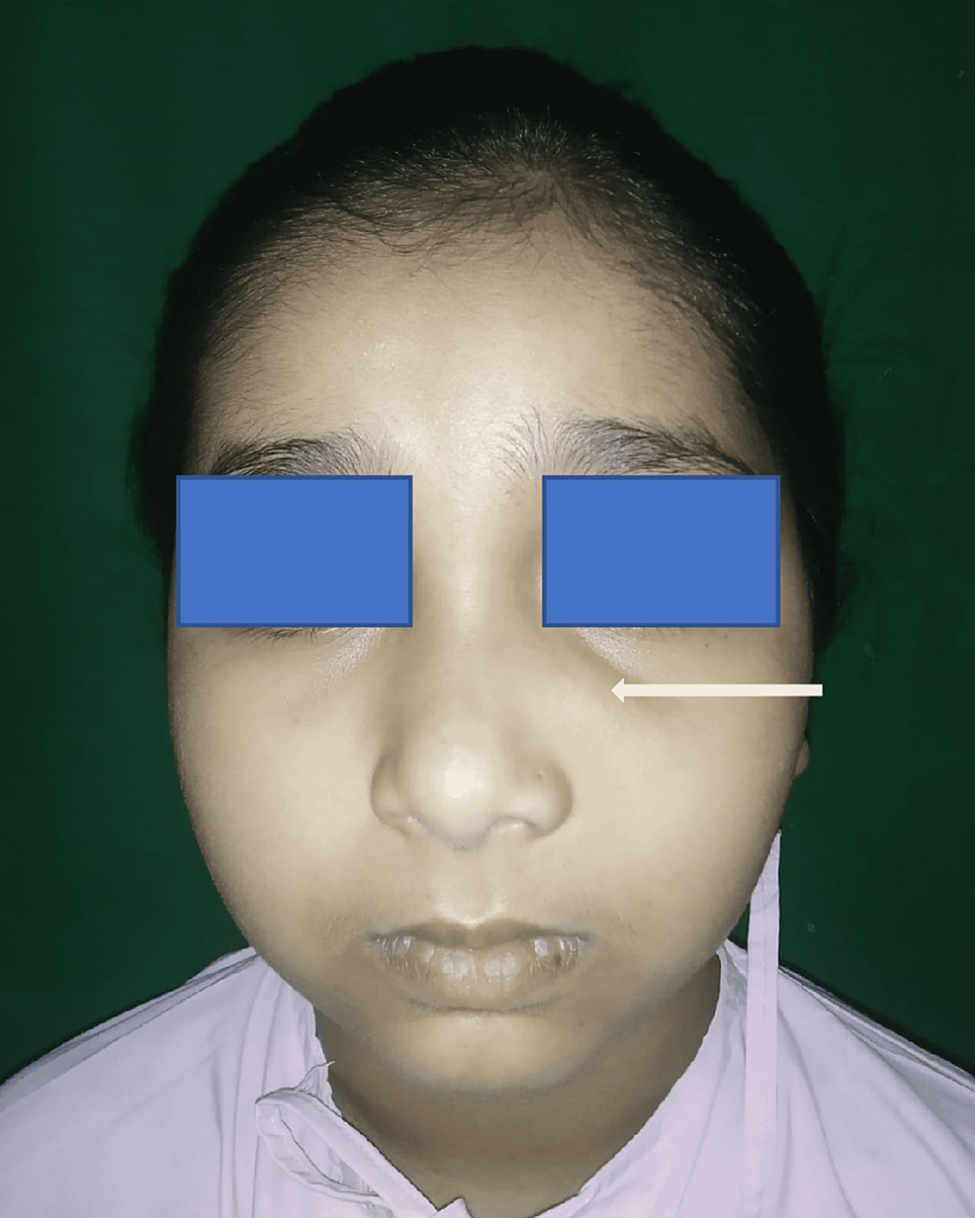
Swelling present over the left maxillary sinus in the nasofacial region.

On examination, the child was asymptomatic. There was evidence of a single, smooth, hard swelling approximately 1x2 cm in size, non-tender on palpation, and not adherent to the overlying skin present over the left maxillary sinus in the left infraorbital region just lateral to the nose in the nasofacial region (Figure 1).

Diagnostic nasal endoscopy revealed, hypertrophied left inferior turbinate with fullness of the left inferior meatus and medialization of the medial wall of maxillary sinus touching the septum posteriorly. Intraoral examination showed no evidence of swelling or missing tooth. Mixed dentition was present with no prior history of trauma, pain, tingling, numbness, or associated lymphadenopathy.

**Figure 2:**
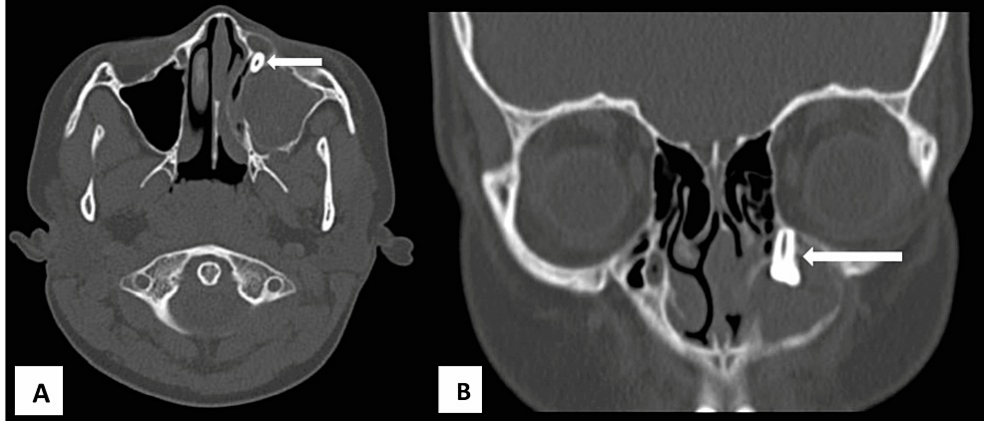
CT, axial (A) and coronal (B) sections, of paranasal sinuses showing a well-defined cystic lesion with an unerupted tooth (arrow) near the anteromedial maxillary wall.

Computerized tomography of paranasal sinuses showed evidence of non-enhancing well-defined expansile heterogenous cystic lesion filling the entire left maxillary sinus causing thinning of all walls of that sinus with embedded unerupted malpositioned tooth placed near the anteromedial maxillary wall impinging the floor of orbit suggestive of dentigerous cyst. The bony wall of the floor of the orbit was intact (Figures 2A, 2B).

**Figure 3:**
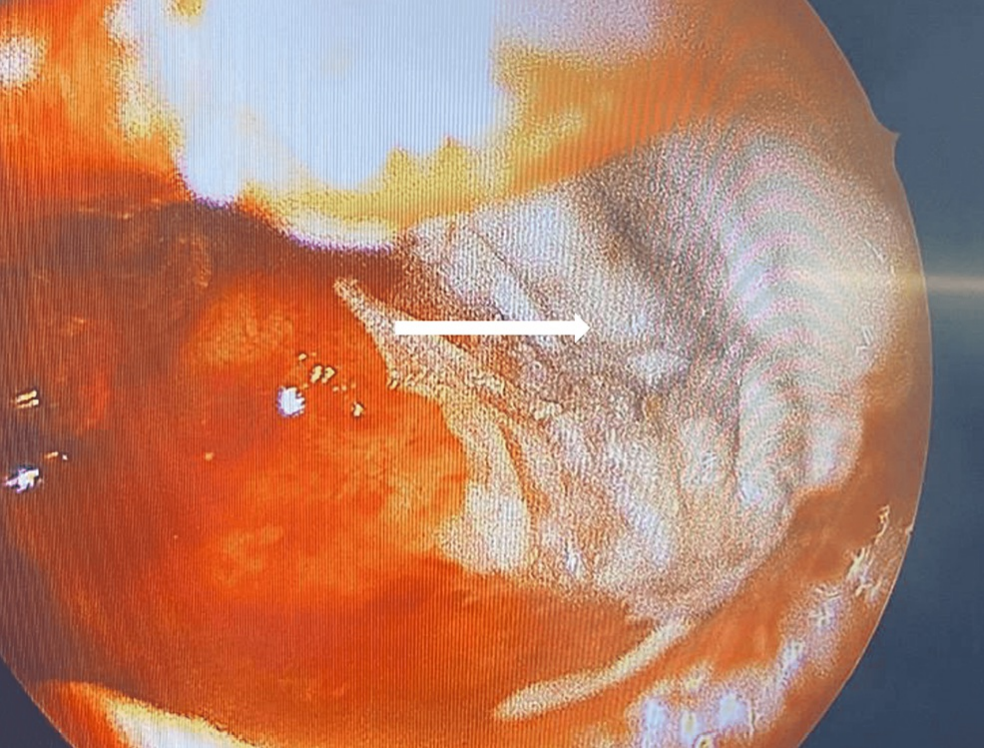
A 30-degree rigid endoscope examination revealed white cheesy material filling the left maxillary sinus.

The child was then planned for endoscopic excision under general anesthesia. Intra-operatively on endoscopic nasal examination, the medial wall of the maxillary sinus revealed fullness and was grossly medialized touching the posterior bony septum. Following medialization of left middle turbinate, uncinectomy was done. The maxillary ostium was widened with a debrider. Examination via a 30-degree rigid endoscope revealed white cheesy material completely filling the sinus (Figure 3).

**Figure 4:**
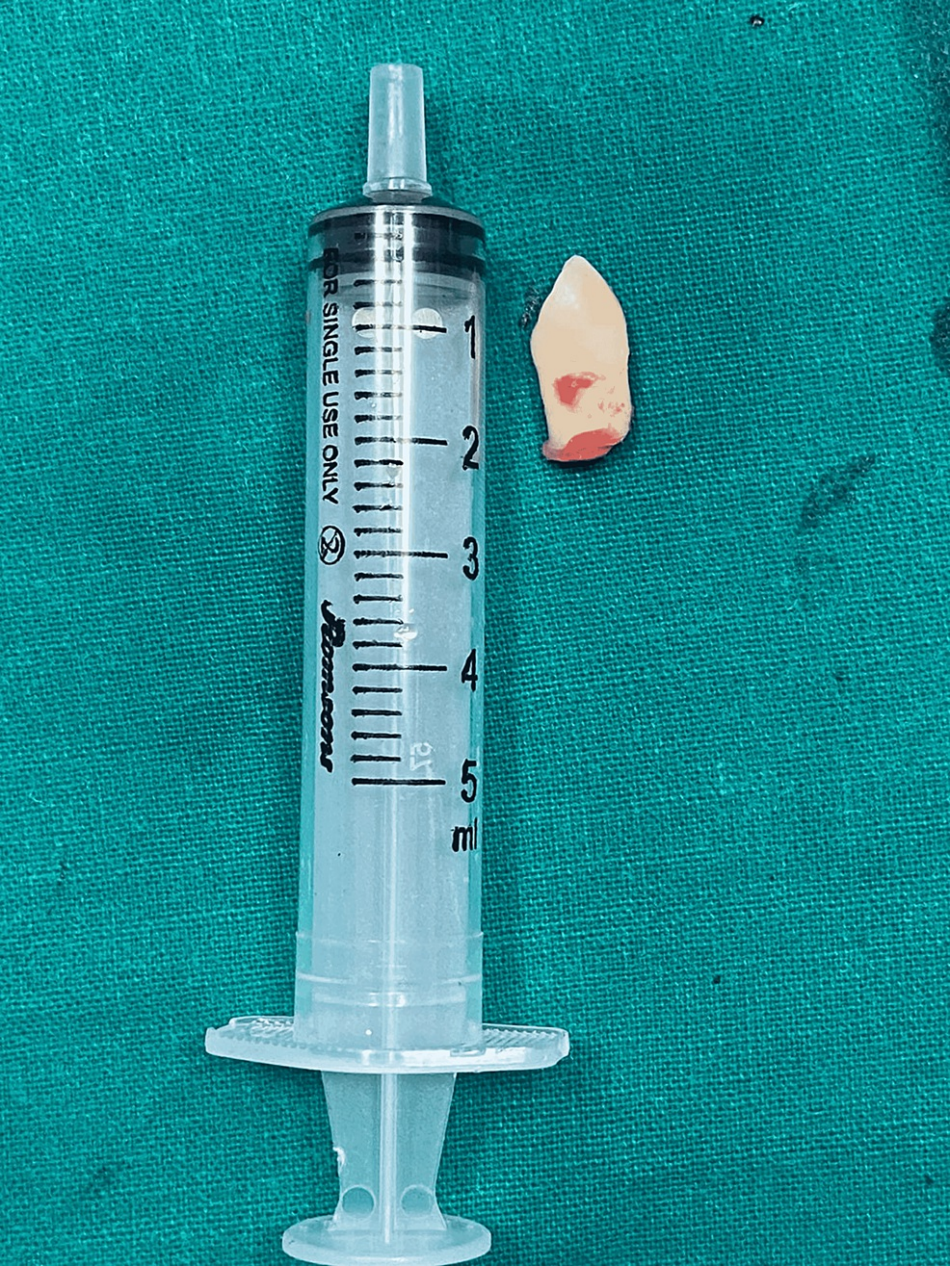
The extracted canine tooth.

It was removed and sent for histopathological examination. Wide marsupialization was performed, in which the medial wall of the maxillary sinus was exposed to ensure that the cyst content was completely drained. Left partial inferior turbinectomy was done posteriorly. Two parallel incisions were taken, first upper incision at the anterior end of axilla of middle turbinate and second lower one, parallel and just above inferior turbinate. Both incisions joined and mucoperiosteal flap elevated anteriorly. The nasolacrimal duct was visualized and a bony incision was given anterior to the duct. There was evidence of tooth placed superiorly near the anteromedial sinus wall. The tooth was removed endoscopically from the sinus through the nasal cavity (Figure 4).

Left lacrimal duct incised with endoscopic scissors, below nasolacrimal sac. Lacrimal sac patency was checked which showed no evidence of obstruction. Histopathological examination of the white cheesy material showed non-odontogenic cyst (keratinizing cyst). The post-operative recovery was uneventful with significant reduction in the size of external facial swelling.

## Discussion

Dentigerous cysts, also known as follicular cysts, are the second most frequent odontogenic cysts after those associated with the roots of the teeth (periapical cysts), originating from the weakened enamel epithelium during tooth crown development. They are most frequently found in the mandible (70%) than the maxilla (30%). The incidence is 14-20% and is slightly more prevalent among males (1.6:1) occurring in the second and third decades of life [1-4]. Uncommon presentation of dentigerous cyst in the female pediatric age group near the anteromedial wall of left maxilla prompted us to publish this case.

A dentigerous cyst is frequently asymptomatic and hence may go undiagnosed for several years [3,4]. It is detected as an incidental radiological finding during an investigation into a failure of tooth eruption, a missing tooth, or mal-alignment, or when secondary acute inflammation, infection, or swelling develops, where it appears as a well-circumscribed, unilocular, usually symmetric radiolucency around the crown of a distinguished radiopaque impacted tooth, usually located on the floor of the maxillary sinus. X-ray of the paranasal sinus (Waters' view), orthopantomogram (OPG), and plain skull radiography are both easy and economical procedures that can be employed in routine clinical settings. The size and extent of the cysts can vary significantly, and ectopic teeth might be located close to the eye; in such circumstances, conventional radiographs may be insufficient for establishing their dimensions or proximity to adjacent vital anatomical structures. Thus, computed tomography (CT) should be used.

An average follicular space is between 3 and 4 mm, when it exceeds 5 mm, a dentigerous cyst is anticipated. Dentigerous cysts can grow to a considerable size and an extensive lesion can cause facial asymmetry [5]. A multitude of parameters influences the selection of the surgical approach, which includes the size of the cyst, the extent of its invasion to the adjacent structures, the age of the patient, and the functional and cosmetic significance of the involved impacted tooth. The conventional treatment method is cyst removal and marsupialization via a Caldwell-Luc procedure. Endonasal endoscopic removal is another technique [6].

The extensive literature review undertaken by Marino et al. describes numerous applications of the endoscopic approach for the treatment of dentigerous cysts, with or without ectopic teeth, and reported no associated significant complications or recurrences with these procedures [7]. Emanuelli et al. proposed excision of ectopic tooth that was associated with a dentigerous cyst through endoscopic lower and middle meatotomies [8]. Song et al. described another endoscopic technique for management of maxillary sinus lesions through anterior or posterior nasolacrimal duct approach [9].

Although the Caldwell-Luc procedure gives direct visualization to the maxillary sinus, it is associated with greater risk of complications and morbidities including damage to the sinus mucosa, retraction of the soft tissues of the cheek, oroantral fistula, infraorbital nerve injury, and damage to the nasolacrimal duct [7]. Thus, it is contraindicated in patients below 17 years of age where enucleation and extraction of an extensive cyst through this procedure can also lead to loss of several teeth resulting in functional, cosmetic, and psychological demerits for the children. Recent advances in endoscopic sinus surgery have redefined various indications for the Caldwell-Luc operation. The endonasal endoscopic approach which employs different angled endoscopes is less intrusive and is associated with fewer complications and better preservation of physiological function.

Dentigerous cysts have the potential to grow into painful, aggressive lesions in the context of persistent infection. Persistently enlarging dentigerous cyst may result in alveolar bone widening, tooth displacement, severe root resorption, extension of the buccal and lingual cortex, and pain. If left untreated, possible consequences include the development of cellulitis, deep neck infection, ameloblastoma, mucoepidermoid carcinoma, or epidermoid carcinoma [10].

## Conclusions

A dentigerous cyst of maxillary canine extending into the maxillary sinus is uncommon. The unerupted tooth involved located near the anteromedial wall and impinging the floor of orbit is infrequent in a female belonging to the pediatric age group. All these rare findings coming together in one case makes it a unique clinical situation that was managed endoscopically with complete cyst excision in a minimally invasive manner considering the age of the patient. In our opinion, early diagnosis with both traditional and advanced imaging modalities is crucial to reduce morbidity and avoid more aggressive surgical procedures to lower the risk of complications. A comprehensive endonasal endoscopic technique, ideally via a middle meatal antrostomy, can be used to extract the tooth and cyst in the maxillary sinus.

## References

[1] Cawson RA, Langdon JL, Eveson JW (2000). Surgical pathology of the mouth and jaws. https://books.google.co.in/books?id=18aTOwAACAAJ&source=gbs_book_other_versions.

[2] Ghandour L, Bahmad HF, Bou-Assi S (2018). Conservative treatment of dentigerous cyst by marsupialization in a young female patient: a case report and review of the literature. Case Rep Dent.

[3] Gaillard F, Jones J (2022). Dentigerous cyst. https://radiopaedia.org/articles/1212.

[4] Girish G, Kumar M, Umashankar D, Sharma R, Veeresh M, Bhandari A (2011). Case report dentigerous cyst in maxillary sinus: a rare occurrence. Int J Oral Maxillofac Pathol.

[5] Ko KS, Dover DG, Jordan RC (1999). Bilateral dentigerous cysts - report of an unusual case and review of the literature. J Can Dent Assoc.

[6] Alhashim FY, Almarhoon FS, Alhashim HY, Moumen A (2021). Endonasal endoscopic management of different cases of dentigerous cysts and ectopic teeth. J Surg Case Rep.

[7] Marino MJ, Luong A, Yao WC, Citardi MJ (2018). Management of odontogenic cysts by endonasal endoscopic techniques: a systematic review and case series. Am J Rhinol Allergy.

[8] Emanuelli E, Borsetto D, Brunello G, Sivolellad S (2018). Endoscopy-assisted removal through combined lower and middle meatotomies of an ectopic upper third molar in the sinus associated with a dentigerous cyst. Oral Maxillofac Surg Cases.

[9] Song XC, Sun Y, Zhang H (2011). Endoscopic maxillary sinus surgery through anterior or posterior nasolacrimal duct approach. [Article in Chinese]. Zhonghua Er Bi Yan Hou Tou Jing Wai Ke Za Zhi.

[10] Anjana G, Varma B, Ushus P (2011). Management of a dentigerous cyst: a two-year review. Int J Clin Pediatr Dent.

